# Challenges and ways forward for sustainable weather and climate services in Africa

**DOI:** 10.1038/s41467-024-46742-6

**Published:** 2024-03-26

**Authors:** Benjamin Lamptey, Salah SAHABI ABED, Masilin Gudoshava, Joseph Mutemi, Mary-Jane Bopape, Elijah Adesanya Adefisan, Moudi Pascal Igri, Ibrah Seidou Sanda, Ousmane Ndiaye, Douglas J. Parker, Andrew J. Dougill, Andreas H. Fink, Peter Knippertz, Steve Woolnough, Erik W. Kolstad

**Affiliations:** 1 University of Leeds, Leeds, UK; 2 West African Science Service Centre for Climate Change and Adapted Land Use, Accra, Ghana; 3 National Meteorological Office, Algiers, Algeria; 4 IGAD Climate Prediction and Applications Centre (ICPAC), Nairobi, Kenya; 5 University of Nairobi, Nairobi, Kenya; 6 South African Weather Service, Cape Town, South Africa; 7 Federal University of Technology Akure, Akure, Nigeria; 8 Climate Applications and Prediction Centre for Central Africa (CAPC-AC), Douala, Cameroon; 9 AGRHYMET CCR-AOS, Niamey, Niger; 10 ANACIM, Dakar, Senegal; 11 National Centre for Atmospheric Science, Leeds, UK; 12 NORCE Norwegian Research Centre AS, Bjerknes Centre for Climate Research, Bergen, Norway; 13 University of York, York, UK; 14 Karlsruhe Institute of Technology, Karlsruhe, Germany; 15 National Centre for Atmospheric Science, University of Reading, Reading, UK; 16 Chr. Michelsen Institute, Bergen, Norway

**Keywords:** Climate sciences, Scientific community

## Abstract

Sustainability of African weather and climate information can only be ensured by investing in improved scientific understanding, observational data, and model capability. These requirements must be underpinned by capacity development, knowledge management; and partnerships of co-production, communication and coordination.

Africa is one of the continents most affected by climate change and variability but has a low capacity to adapt^1^. Science that supports a good provision of weather, water and climate services is urgently needed for an adaptation strategy^2^ and will contribute towards resilience. Delivering such services will address the March 2022 call by the UN Secretary General for “Early Warnings for All” within 5 years^1^. Currently we are being held back by a key problem; that the massive improvement in global weather and climate science and services, and their role in environmental and socio-economic welfare benefits, has not been reflected in Africa as it has in other parts of the globe^3^.

In this article we highlight four challenges. Mostly, these have been known for many years, but solutions have been slow to come and overcoming such long-standing problems will need new incentives that will facilitate to achieve such solutions. We propose such incentives for each challenge, through approaches integrated into existing structures. We consider solutions to all of these interconnected challenges^4^ to be *necessary*, and therefore the order of these is not in terms of importance.

## Challenge 1: scientific gaps

African weather and seasonal forecasting are held back by gaps in our understanding of the underlying science of the meteorological processes in the tropics^1^. Furthermore, the African research community lacks sufficient scientific capacity and capability in this field^3,5,6^. Without African ownership of scientific advances, the continent will always be reliant on externally-imposed solutions and not able to address scientific challenges that may be unique at the community scale.

Tropical weather systems over Africa, which differ from mid-latitude systems^7^, have not been studied well enough to support all aspects of operational weather prediction. The lack of proper understanding of these systems has led to their poor representation in numerical weather prediction (NWP) models, resulting in poor performance, especially for rainfall^4,8^. Our limited understanding is exacerbated by lack of scientific specialists, and university programmes on the continent remain limited^9^. Many scientists have insufficient access to necessary resources, such as supercomputing systems.

Incentive: We believe the solution is to invest in advanced quantitative and qualitative training in the core sciences, which underpin data analysis^10,11^ and weather and climate forecast models^7,12,13^. We need to support long-term scientific careers within Africa with adequate research infrastructure in the form of computing, models and data facilities. This should be done by strengthening African research capability around existing centres of excellence with top-level scientific infrastructure and a clear vision for growth. International aid and development funding needs to prioritise scientific centres of excellence and long-term African career paths in climate sciences. The progress and impact of existing centres of excellence serves as incentive to implement the suggested solutions. The West African Science Service Centre for Climate Change and Adapted Land Use (WASCAL) programme^14^ demonstrates how international investment has led to substantial growth in capacity in climate science since the centre was established in 2011.

## Challenge 2: data gaps and data access

We need to improve past, present and future observations^15,16^. This need has been expressed for decades, but not enough has been accomplished. Data rescue is needed for the non-digitized data. The observations are needed for improved scientific understanding (see Challenge 1); model verification, development and tuning; data assimilation; postprocessing or bias correction and the development of statistical and machine learning forecast approaches to replace or complement model-based forecasts.

Investing money is not sufficient to tackle this decades-old problem. Many millions of capital funding have been spent on the delivery of weather radars to African countries, but very few of them deliver useful data^17,18^, due to a lack of operational investment^16^. But there are also models of success. The African Monsoon Multidisciplinary Analysis Project (AMMA)^2^ demonstrated that the observing network can be delivered to high international standards by African agencies^3^, if management is able to deploy financial resources tactically and strategically to support infrastructure on the basis of success in data collection.

A known challenge is the difficulty in obtaining data from the National Meteorological and Hydrological Services (NMHSs). The discussion and the need for advocacy for easy access to the available data that are not easily accessible, has been going on for a long time.

Incentive: Learning from the AMMA experience, a coordinated African approach with support from the recent World Meteorological Organization (WMO) Systematic Observations Financing Facility (SOFF) initiative^19^, is a new incentive for an African solution to an African challenge. SOFF promotes reliable and accurate data collection because its financial support is contingent on the successful delivery of data to international servers, monitored long-term.

SOFF also therefore encourages free exchange of data, by incentivizing the delivery of data to open data repositories.

## Challenge 3: modelling and forecasting gaps

Delivery of weather and climate services is held back by the low accuracy of models over Africa, leading to high uncertainty in the prediction of rain-bringing deep convection, particularly over time scales of a day or so^7,20,21^. Most global weather and climate models were developed in and for the mid-latitudes and we urgently need to apply different strategies to how the models are run and exploited for Africa.

There are several connected reasons for the poor performance of models for daily African rainfall. The inherent chaos of convective systems, and thus low predictability of rainfall in the tropics^7,20^, is particularly severe in Africa^8^. Low intrinsic predictability warrants the use of ensemble strategies, which themselves need to be calibrated with better observations. The longer predictability at larger scales in the tropics implies that statistical-dynamical modelling approaches (including machine learning) hold some promise and there are windows of opportunity for flow-dependent forecasts that are not yet being exploited. Advances in high performance computing and observing systems that have contributed to the progress in numerical modelling globally, are lagging behind in Africa^8^.

To improve model performance, we need to improve the handling of tropical processes and weather systems in the models, advance ensemble modelling, and apply convection-permitting models (CP) with an “African lens”^21^. For improved models to benefit African end users, exploitation of real-time observational data through improved data assimilation will be vital^10^, also leading to benefits in climate analysis and in validating global models^22^.

At sub-seasonal and short time scales, there is potential skill in some global models that can be utilised if access to forecast products from the international producing centres^11^ could be granted. Combined statistical-dynamical modelling including machine learning, offers opportunities to improve the skill of the data provided over tropical Africa^23^ to end-users. Socio-economic and impact-based evaluation of models needs to be led and owned by Africans researchers and end-users and not imposed from outside. Recognising the limitations on prediction of tropical convection due to chaotic atmospheric developments, we have to improve “nowcasting” of existing storms over Africa^5,24^.

There is the need to enhance cooperation between universities and national forecasting services or National Meteorological and Hydrological Services (NMHSs) in both the Global South and North. We have to co-design modelling solutions (including co-designing of NWP models in cloud-based computational platforms), to find the best balance between technical feasibility, sustainability and robustness on one side and sufficient acceptance, ownership and freedom of configuration on the other hand.

Incentive: International donors have to ensure that African scientists have skills and facilities to co-produce^25,26^ and deliver services that reach communities and stakeholders in every country and region. Centres of excellence, in particular the African universities and WMO Regional Training Centers (RTCs), can train large numbers of specialists to work across diverse countries and communities, as has been achieved by WASCAL^14^. Investment in High Performance Computing, NWP and Climate Modelling within a number of leading African Centres is required, to build local capability, communities of expertise and ensure feedback to Global Centres. These benefit science globally and motivate African Scientists to continue their work in the region.

## Challenge 4: capacity building, knowledge management and communication

Major international programmes such as Climate Risk for Early Warning Systems (CREWS)^27^ and Weather and Climate Information Services (WISER) are putting significant financial and organizational resources into the challenge of improving African EWS. Tackling Challenges 1–3 within a few years will require us to strengthen management, governance and communication of regional activities, to increase capacity and exploit learning across many stakeholders and projects. The scale of the challenge is global and across many disciplines with investments in early warnings and actors especially vital for saving lives, promoting economic development and reducing the cost of disaster responses in an African context^28^. Although national and international coordination mechanisms and agencies exist, there are still too many examples where operational systems fail when agencies fail to cooperate^3^.

We need to enhance coordination for the optimal use of technology and optimal network of observing and communication systems, including training a critical mass of human capacity to support these systems. Existing coordination in these areas is overseen by the WMO^29^, but the WMO lacks direct resources, and can mostly only provide oversight to strategy. Future coordination will be strengthened, if it is backed by performance-based funding, as in the SOFF initiative^19^. Capacity building should be done within a framework that ensures the concept of “Training of the Trainers (TOT)”. Investments to support the transition from research to operations, partnerships between universities WMO RTCs and NMHSs that strengthen the provision of skills across the range of disciplines from physics, computational science through to social and economic sciences, need to be part of the solutions.

But long-term sustainable solutions also require us to address the reliance of the Global South on the Global North and the unequal dynamic between producers and users within the Global South^30^. Additionally, the co-production of climate services at regional and national level within the Global South is still low and this is reducing the uptake of climate information and its mainstreaming into decision making processes^31,32^. Effective partnerships must entail knowledge exchange, capacity building and co-production to address the unequal dynamic among actors, including the African Centre of Meteorological Applications for Development (ACMAD), the Regional Climate Centres, African universities and WMO RTCs^33^

Sustainability is also linked to innovation and job creation. Young professionals crave exciting job opportunities, and if African countries are successful in educating students with expertise in weather and climate services, these students will be empowered to create new products and services which are useful for farmers and businesses. Such products are needed, and job creation is also hugely important for African countries with a young and forward-looking population. However, although the African weather services market is estimated at around $60 million currently, none of the top 20 public and private sector providers are based in Africa^16,34^.

Incentive: To ensure a balance in partnerships that will prevent solutions being imposed from outside, donors and programmes^27^ need to insist, right from the start, on standards of governance for international coordination, and to incentivise African leadership of projects, with funding linked to successful outcomes. The WMO Multi-hazard Early Warning System (EWS) best practice value chain, if implemented appropriately, could be a blueprint for co-production because of the needed input from both the Global South and North. The chain is “(1) Knowledge, (2) Forecasting, (3) Communication (Dissemination and Feedback), (4) Preparedness and (5) Monitoring and Evaluation (including socio-economy)^21^.

## Outlook

The Early Warnings for All and SOFF initiatives, are responding to recognition that African weather and climate services have failed to keep up with global progress in recent decades. But it will not be sufficient to simply impose solutions which work in the Global North: increasing the availability of inaccurate forecasts from NWP will not give people better early warnings; sending more radars to Africa will have no impact on data availability unless we invest in local capacity and capability to maintain and exploit them. We recommend the implementation of a number of incentives, to motivate the solutions that will address long-standing challenges to African weather and climate science and services within the shortest possible time. In particular, funding bodies need to demand standards of partnership, governance and African leadership; to prioritise long-term investment in scientific infrastructure and careers; and to link long-term support more directly to successful delivery. Overcoming these challenges will allow African agencies and communities to properly benefit, and contribute to, the advancement in global weather and climate science and services, and their role in environmental and socio-economic welfare.
